# Exploring ableism and occupational therapy: Occupational therapy students’ perspectives

**DOI:** 10.1177/03080226241309469

**Published:** 2025-01-16

**Authors:** Hannah Darton, Alison Wadey, Alison Laver-Fawcett

**Affiliations:** 1 Tees, Esk and Wear Valleys NHS Trust, York, UK; 2 York St John University, York, UK

**Keywords:** Ableism, occupational therapy, disability studies, students, survey

## Abstract

**Aim:**

To explore occupational therapy students’ perspectives on ableism and its implications for occupational therapy practice. This formed part of a wider study that also explored occupational therapy educators’ perspectives.

**Method:**

An online survey was used to collect students’ perspectives, using a mixture of Likert scales and open-ended questions.

**Findings:**

The sample comprised 56 occupational therapy students from the United Kingdom (*n* = 36), United States of America (*n* = 16) and Canada (*n* = 4) enrolled in a mixture of undergraduate (*n* = 13) and postgraduate (*n* = 43) pre-registration degree programmes. Thirty-four percent of respondents perceived occupational therapy as inherently ableist. This rose to 50% after respondents were presented with a comprehensive definition of ableism. Students reported witnessing and/or experiencing ableism within education (63%) and practice placements (55%). Eighty-six percent of students recognised they may hold unconscious ableist views, and 96% agreed they would like more support to engage in disability studies.

**Conclusion/Impact:**

Findings indicated a potential link between understanding of ableism and students’ views that occupational therapy is ableist. Most students were aware of the potential they hold unconscious biases and welcomed support to engage further with disability studies. Further qualitative research is needed. Following this, systemic changes to address the harm of ableism can begin to be addressed.

## Introduction

The occupational therapy profession has long navigated a complex identity (Turner and Knight, 2015), intertwining its fundamental belief in the inherent connection between occupation and health (Wilcock and Hocking, 2015) with external pressures to define, measure and validate its role within healthcare systems (Fitzgerald, 2014). This dynamic interplay has been shaped by various factors, including regulatory demands and institutional forces, often framed within the context of neoliberal, capitalist societies that prioritise productivity and independence (Grenier, 2020). Consequently, occupational therapy scholars and practitioners have increasingly called for critical reflection on how these societal values influence the profession and potentially perpetuate harm (Hammell, 2022; Karp and Block, 2022; LeBlanc-Omstead and Mahipaul, 2022; Mahipaul, 2022; Tsang and Haque, 2022; Vine, 2024; Yao et al., 2022).

This article responds to this call for reflection by focusing specifically on the intersection of ableism and occupational therapy from occupational therapy students’ perspectives. Utilising a survey, the research sought to explore the perspectives of students enrolled in occupational therapy degree programmes, aiming to shed light on their understanding of ableism, experiences of ableism within the field, and perceptions of its impact on occupational therapy education and practice. Through this exploration, the authors endeavour to contribute to a deeper understanding of the complex dynamics surrounding ableism in occupational therapy.

## Literature review

### Ableism

The concept of ableism, as defined by the Oxford English Dictionary (2023), denotes ‘discrimination in favour of able-bodied people; prejudice against or disregard of the needs of disabled people’. However, this definition has been critiqued for its simplicity and the ambiguity surrounding the term ‘able-bodied’. Disability scholar Fiona Campbell (2009) proposed an alternative perspective, framing ableism not solely as discrimination but as a complex network of beliefs, processes and practices. Similarly, Lewis (2022) adopted an intersectional approach, highlighting the underlying roots of ableist ideology, they define ableism as ‘*A system of assigning value to people’s bodies and minds based on societally constructed ideas of normalcy, productivity, desirability, intelligence, excellence, and fitness. These constructed ideas are deeply rooted in eugenics, anti-blackness, misogyny, colonialism, imperialism, and capitalism. This systemic oppression leads to people and society determining people’s value based on their culture, age, language, appearance, religion, birth or living place, “health/wellness”, and/or their ability to satisfactorily re/produce, “excel” and “behave”. You do not have to be disabled to experience ableism*’. This shift aligns with a broader call within the disability justice movement to move beyond mere disability rights towards a more comprehensive understanding of disability justice (Mahipaul, 2022).

### Ableism and occupational therapy

Despite longstanding calls for reflection on the incorporation of disability studies within occupational therapy education (Linton, 1998), the field has only recently begun to engage in critical discourse on ableism. Notably, *The American Journal of Occupational Therapy* (AJOT) published a special issue on disability studies (DS) in 2005 (Kielhofner, 2005). A follow-up special issue interviewed eleven of the original authors, concluding that while their engagement with disability studies had impacted their own practices, there remained little overlap within wider occupational therapy scholarship (Harrison et al., 2021; Sheth et al., 2021) .

Research on ableism within the profession is often in the form of critical reflections (Hammell, 2022; Karp and Block, 2022; LeBlanc-Omstead and Mahipaul, 2022; Mahipaul, 2022; Tsang and Haque, 2022; Yao et al., 2022). Some research has examined implicit and explicit ableist biases among occupational therapy students and professionals (VanPuymbrouck and Friedman, 2019; Friedman and VanPuymbrouck, 2021a, 2021b; Feldner et al., 2022). These studies all used the Disability Attitude Implicit Association Test (DA-IAT), a tool gauging implicit disability attitudes by measuring response times to associations between disability and positive/negative attributes (Project Implicit, 1998). Their findings revealed implicit biases towards disabled individuals among occupational therapy students and professionals. Despite the DA-IAT’s established validity and high test-retest reliability (Aaberg, 2012; Pruett and Chan, 2006; Thomas *et al.*, 2014), it is a single measure focused on individuals rather than taking a systems approach (Grenier, 2020).

Additionally, current published research has primarily emerged from North American, Canadian, and Scandinavian contexts (e.g., Harrison et al., 2021; LeBlanc-Omstead and Mahipaul, 2022; Mahipaul, 2022; Tsang and Haque, 2022). There remains a scarcity of literature, particularly from the United Kingdom (UK), exploring ableism within occupational therapy education. The emergence of initiatives like ABLEOT UK (ABLEOT UK, 2021), a UK-based occupational therapy network and advocacy group, highlights the increasing need for practitioners, students, researchers, educators, and individuals with disabilities or long-term health conditions to support one another and address ableism within the profession. By offering resources and support meetings on a variety of topics, ABLEOT UK underscores the importance of occupational therapists critically reflecting on their practices and engaging with disability studies (Hicks, 2022).

This literature review highlights the imperative for occupational therapy education to confront and challenge ableist narratives. This study aimed to contribute to the evidence base and build on previous work by exploring nuances in occupational therapy students’ understanding of ableism and its manifestations in occupational therapy.

## Methodology

This article constitutes the first part of a study investigating perspectives within occupational therapy degree-level education concerning ableism and its implications in occupational therapy practice. This article solely focuses on presenting findings from the student sample, while the second part of the study, published separately, addresses teaching staff responses. The study utilised a survey to collect both qualitative and quantitative data. A single online survey, incorporating branching logic, was used across both segments of the study. The survey comprised four distinct sections:

Part 1: Dedicated to gathering demographic data and details regarding respondents’ educational backgrounds.
Part 2: Consisted of seven statements with a 5-point Likert scale (strongly agree, somewhat agree, neither agree or disagree, somewhat disagree, strongly disagree) with space for additional comments. These statements aimed at eliciting insights into respondents’ perspectives on current occupational therapy practices.
Part 3: Featured one open-ended question to gauge respondents’ comprehension of ableism, along with two Likert questions, supplemented by space for comments. This survey section aimed to ascertain respondents’ views on whether occupational therapy perpetuates ableism, both before and after exposure to Lewis’s (2022) definition of ableism. By obtaining students’ opinions prior to sharing the definition of ableism, the researchers aimed to capture their unfiltered perceptions and identify any preconceived notions they may have about the term.
Part 4: Comprised of seven statements with a 5-point Likert scale (strongly agree, somewhat agree, neither agree or disagree, somewhat disagree, strongly disagree) with space for additional comments. This section aimed to delve into respondents’ experiences with ableism and their interactions with disability studies. By providing the definition of ableism prior to these questions, researchers aimed to encourage students to consider their experiences in relation to this definition of the concept.

The survey was constructed using Qualtrics (Qualtrics XM, 2022). Purposive and snowball sampling techniques were employed to recruit participants. The survey was disseminated via posts on Twitter (now X), briefly explaining the study and with an embedded link to the study information and survey. The post encouraged people to repost the link, and it was shared 68 times. Emails with a brief explanation of the study and a link to the survey were sent to course leaders of UK-based occupational therapy degree programs with a request to share with their students. Responses were collected over a 25-day period during spring 2023. Inclusion criteria were students enrolled in World Federation of Occupational Therapists (WFOT)-accredited occupational therapy programs worldwide who were proficient in English. Due to resource limitations, participants were required to be able to complete the study materials in English without the need for translation services. The inclusive criteria were designed to capture diverse perspectives suitable for an exploratory study (Denscombe, 2021). Data analysis involved descriptive statistics to outline participants’ demographics, experiences, and Likert-scale responses. Additionally, content analysis, used in similar research (VanPuymbrouck and Friedman, 2019), was employed to explore students’ definitions of ableism.

Ethical approval was obtained from York St John University Ethics Board. The study involved administering an anonymised survey to a non-vulnerable group. An information sheet outlining the survey’s objectives, data handling procedures, and participant rights was provided on the landing page of the online survey to ensure transparency. Although participants were not classified as vulnerable, the study explored themes surrounding potential discrimination towards disabled individuals; therefore, participants were informed about avenues for lodging complaints and where they could seek support. Explicit consent was gained once participants had read the pre-information and prior to them beginning the survey.

The survey was co-designed by two of the authors, the first author was a master’s student, and the second was a senior lecturer on a UK-based pre-registration occupational therapy master’s degree program. The survey was additionally piloted by two further occupational therapy students, one based in the United Kingdom and one from the United States of America. Analysis of the results was overseen by the third author, a professor in occupational therapy at a UK-based university. Including additional researchers at various levels of occupational therapy expertise helped to mitigate researcher bias. Continuous researcher self-reflection and meticulous documentation of data collection and analysis processes further contributed to the study’s dependability and transferability.

## Results

### Respondents’ demographics

The online survey gathered 56 valid responses from occupational therapy students who met the inclusion criteria, provided consent and completed at least 80% of the survey. Henceforth, references to ‘respondents’ pertain to the occupational therapy student participants. Respondents were given a participant code, S for a student plus a number, with codes from S1 to S56. The relevant code has been provided alongside any direct quotes from the sample’s responses. Please refer to Table 1 for a breakdown of respondents’ demographics.

**Table 1.** Respondents’ demographics and experience.

Total respondents	Total count (%, rounded to 1 decimal place)
	*N* = 56
Gender	
Male	3 (5.4)
Female	52 (92.9)
Non-binary	1 (1.8)
Disability identity	
None	16 (28.6)
More than one	17 (30.4)
Specific Learning Difficulty (SpLD), Neurodivergent,	6 (10.7)
Mental illness/disability	12 (21.4)
Physical illness/disability	4 (7.1)
Prefer not to say	1 (1.8)
Age (years)	
Mean (standard deviation):	30 (8.1)
<21	4 (7.1)
21–30	33 (58.9)
31–40	11 (19.6)
41–50	8 (14.3)
51–60	0
61–70	0
Country of current education institute	
Canada	4 (7.1)
United Kingdom	36 (64.3)
United States of America	16 (28.6)
Degree level *n* (%)	
Bachelors	13 (23.1)
Masters	28 (50.0)
Doctorate	15 (26.8)
Year of study	
One	24 (42.9)
Two	20 (35.7)
Three	8 (14.3)
Four	3 (5.4)
Five	1 (1.8)
Clinical Experience (students with at least one placement experience) *n* (%)	
Adult	49 (88)
Physical	37 (66)
Community	22 (39)
In-patient	29 (52)
Mental	34 (61)
Community	28 (50)
In-patient	12 (21)
Paediatrics	16 (29)
Physical	15 (27)
Community	11 (20)
In-patient	5 (9)
Mental	6 (11)
Community	5 (9)
In-patient	1 (9)

The gender distribution among respondents was 5% male, 93% female and 2% non-binary. Over half (61%) of respondents identified as having a disability, health condition, specific learning disability, or as being neurodivergent. The average age of respondents was 30 years old. With the majority (59%) of respondents falling into the 21–30 age group. Respondents ages ranged from 19 to 49 years old. All of the respondents’ educational institutions were located within the Global North: United Kingdom (64%), United States of America (29%) and Canada (7%). Respondents were enrolled in a mix of degree type, with the majority (50%) enrolled in master’s level programmes. Only one respondent was in their fifth year of study while 43% were in their first. More students had experience in adult clinical settings (88%) than in paediatrics (29%). Only one student had only experience of paediatrics with no adult services placements. Six respondents had no placement experience.

### Occupational therapy processes, procedures and values

**Figure 1.**
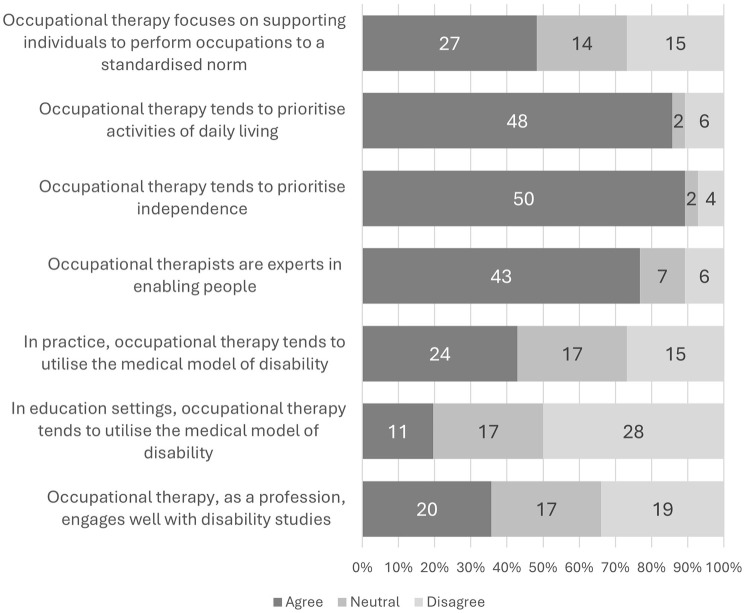
Respondents’ views on occupational therapy (*N* = 56).

This section of the survey investigated if the elements of occupational therapy that Hammell (2022) identified as potentially contributing to ableism aligned with the respondents’ experiences. Students evaluated seven statements pertaining to occupational therapy practice using a 5-point Likert scale with options including ‘strongly agree’, ‘somewhat agree’, ‘neither agree nor disagree’, ‘somewhat disagree’ and ‘strongly disagree’. To simplify the analysis and presentation of results, at times the responses that indicated a positive level of agreement (‘strongly agree’ and ‘somewhat agree’) were categorised as ‘agree’, while those indicating a negative level of agreement (‘somewhat disagree’ and ‘strongly disagree’) were categorised ‘disagree’. The findings are illustrated in Figure 1.

#### The focus of occupational therapy

Hammel’s (2022) critique examined four aspects of occupational therapy. Hammel argued that occupational therapy often overemphasises independence, neglecting the needs of individuals who may require ongoing support. Additionally, Hammel criticised occupational therapy for primarily focusing on activities of daily living (ADLs), while overlooking other important aspects of occupational engagement, such as leisure, work and education. Furthermore, Hammel suggested that occupational therapists often view themselves as experts who enable individuals to participate in activities, rather than collaborating with clients as partners. Finally, Hammel argued that occupational therapists frequently prioritise standardised measures of performance, which can overlook individual differences and unique experiences. Respondents were asked to what degree they agreed that these four areas are a focus of occupational therapy. Students agreed with an emphasis on independence (89%) and a focus on ADL (86%). They also saw therapists as expert enablers (77%). Interestingly, only 48% agreed with prioritising standardised performance. Fifty percent of the 26 respondents, who provided additional information to the statement relating to occupational therapists being expert enablers, highlighted the importance of recognising the expertise of those who were engaging in occupational therapy services.

#### Use of the medical model of disability

While only 20% of respondents agreed the medical model is used in occupational therapy education, 43% agreed it is present in practice. This trend held true for both the United Kingdom (17% in education, 44% in practice) and North America (25% in education, 40% in practice).

#### Occupational therapy and disability studies

Respondents’ opinions were divided regarding occupational therapy’s engagement with disability studies, with 36% agreeing, 30% neutral, and 34% disagreeing. Interestingly, agreement was higher in North America (50%) compared to the United Kingdom (28%). Students with disabilities, neurodivergent conditions or long-term health conditions showed similar agreement levels (35%) to those without (38%). However, a significant disparity in disagreement levels emerged, with 43% of students with disabilities, neurodivergent conditions, or long-term health conditions indicating disagreement, compared to 13% of students without such conditions.

### Understanding ableism and perspectives on the link between occupational therapy and ableism

#### Is occupational therapy ableist?

This section of the survey investigated views on occupational therapy and ableism. Only 34% of respondents agreed that occupational therapy is ableist, while 29% were neutral and 38% disagreed. Importantly, those who identified as disabled, neurodivergent or with a health condition were more likely to perceive ableism (40%) compared to those without (19%). There was a large degree of disparity in levels of agreement that occupational therapy is ableist between those still in their first year of study (21%) and those post their preliminary year (44%).

The most common reason for agreeing with the ableism label was the profession’s emphasis on ‘normal’ function and standardised assessments (47%): ‘*Many standardised assessments and interventions work towards goals that do not always consider lives, desires or relationships outside of the standardised norm*’ (S21). Disagreement reasons varied: nearly half (47%) simply disagreed without further explanation. However, some explanations were provided, for example: ‘*I feel it contradicts everything we stand for*’ (S17). Others highlighted occupational therapy’s focus on empowerment (10%), ‘*Occupational therapists do not discriminate their clients. In fact, they empower them*’ (S49), its dependence on individual practitioners (10%), or pressures within specific practice areas (10%), ‘*OTs can get caught up in the medical model that is present in many settings*’ (S31).

#### Respondents’ understanding of ableism and their perception of occupational therapy as ableist

Most respondents (all except one) defined ableism in their own words. These definitions were analysed and categorised into four main definition types, with the percentage of students who used each definition type shown in brackets:

1. Socially constructed ‘normal’ (20%): This group focused on the idea of a forced conformity to a societal definition of ‘normal’.
2. Disabled people are deficient (9%): This group focused on the view of disability as a negative state that needs fixing or curing.
3. Discrimination only (67%): This group solely focused on discrimination against disabled people.
4. Incorrect definition (4%): This group provided definitions that did not align with published definitions of ableism in the reviewed literature.

After being introduced to Lewis’ (2022) definition of ableism, most respondents (93%) reconsidered their initial answer to ‘Is occupational therapy ableist?’. The majority (60%) maintained their original opinion. However, 31% increased their level of agreement with the statement, while 10% increased their level of disagreement. Notably, only one person shifted from agreement to disagreement. The remaining four respondents who decreased their level of agreement moved from strong agreement to either weaker agreement or neutrality.

Respondents were asked to explain their reason for the level of agreement after being provided with a definition of ableism within the survey. Sixty-three percent of respondents who increased their level of agreement with the statement provided explanations regarding their new level of agreement rating. Themes emerging from these explanations included: influences on occupational therapy by broader societal norms and expectations; the profession’s roots in Western culture and, therefore, its limited ability to address diverse needs of individuals; the profession’s focus on ‘normal’ function and productivity; and systemic issues within the broader healthcare system. Three of the six respondents who lowered their level of agreement to the statement offered explanations: one commented *‘I don’t know’* (S23); another commented *‘Not great support for disabled workers’* (S7); the third stated *‘There’s a difference between what OT preaches and OT in practice. In theory - we are not ableist, in practice we are’* (S45).

### Reflections on ableism and occupational therapy

**Figure 2.**
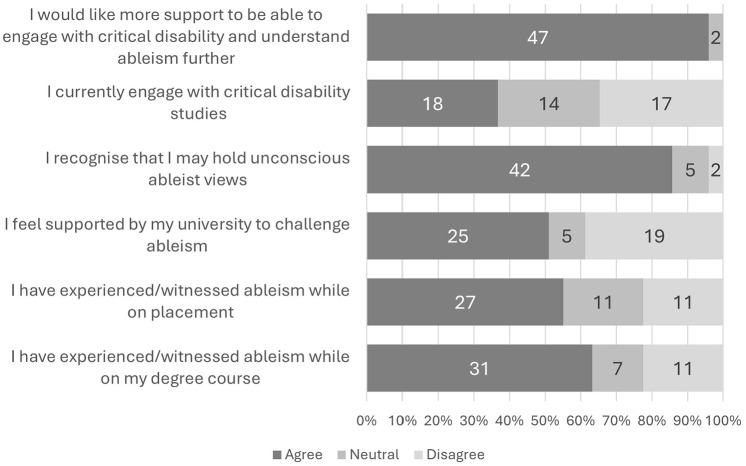
Respondents’ reflection on occupational therapy and ableism.

This section of the survey was designed to explore respondents’ perspectives and experiences with ableism within occupational therapy. Figure 2 illustrates the outcomes of Likert responses to six statements (note that one statement has been removed from the analysis. See limitations section for explanation). The response rate for these statements was 88% (*n* = 49).

#### Witnessing and challenging ableism

A significant proportion of students reported encountering or personally experiencing ableism during their occupational therapy education (63%) and on placements (55%). Just over half (53%) of the students who responded to these two Likert questions added written explanations for their responses. Comments relating to educational settings commonly related to witnessing ableist behaviour from lecturers (23%); ‘*professors making statements like ‘autistic people have no perspective’ or repeatedly referring to people who use substances as “nasty”’* (S41), and ‘*mobility aids have been described as ugly and out of the norm*’ (S11). Twelve percent mentioned witnessing other students’ behaving in ableist ways, but no examples were given. Thirty-eight percent mentioned experiencing or witnessing reasonable adjustments such as ‘*turning off or dimming lights, providing lecture slides before sessions*’ (S39) were not being met. One student spoke of how their adjustment needs were ‘*denied by the lecturer as they stated it would be unprofessional for these needs to be met and that my employer would not allow for these needs to be met*’ (S38).

In placement settings, 23% percent of comments spoke of how the views of the individuals within services were not being considered, such as refusing to perform a wheelchair assessment ‘*because it was raining and they assumed the individual wouldn’t want to get wet*’ (S20), or prioritising that a patient ‘*remain living at home as independently as possible even if this was not what the patient or their family wanted*’ (S37). Additionally, 19% of comments related to witnessing inappropriate language being used in practice, often in general terms ‘*stigmatizing language*’ (S40) and ‘*off handed comments*’ (S44) but some provided more specific examples. For example, ‘*patients being called “crazy”*’(S31).

Roughly half (51%) of students agreed they felt supported by their universities to challenge ableism. Respondents’ explanations for the level of support they felt varied greatly, one wrote ‘*I feel that ableism is not tolerated at [Name of institution redacted for anonymity] and I feel supported by them to challenge any bigotry*’ (S30). While others made accusations of *‘gaslighting [from] unsupportive administration’* (S55) and were concerned challenging ableism *‘would impact on my marks as other students who challenged them failed their assignments’ (S16).*

A disparity in perceived levels of support to challenge ableism existed between students with disabilities, long-term health conditions or neurodivergence (44% agreed) and those without (69% agreed). Additionally, a gap emerged in perceived support to challenge ableism between students in the United Kingdom (33%) and those in North America (79%).

#### Recognising ableism and engagement with disability studies

Results showed 86% of respondents agreed that they are aware they may hold unconscious ableist biases and only two respondents (4%) disagreed with the premise of holding unconscious ableist biases. One student (S26) who disagreed still recognised the ongoing challenge of overcoming internalised societal views, stating, ‘*dismantling internalised societal views is a constant work in progress*’. The other student (S17) referenced their personal experience as a caregiver for someone with significant disabilities as the reason they believed they were not susceptible to such biases.

Students’ engagement with disability studies showed mixed responses. Around 37% agreed they currently engage, while 35% disagreed, and the rest remained neutral. Notably, students with disabilities, neurodivergent conditions, or long-term health conditions were more likely to already be engaged (42%) compared to those without (23%). However, a strong interest in further engagement emerged across the sample. A resounding 96% of respondents agreed they desired to learn more about disability studies, with only two students remaining neutral and none disagreeing. Only 25 students added comments to the open text box for this Likert statement and 10 of these were simply reiterating their agreement. Of the remaining 15 who responded with reasons why they agreed with the statement, comments related to it being ‘*essential to moving forward as a profession’* (S9) as a whole, as well as more personal reflections on how it would support their own practice ‘*I feel this would be beneficial for my future practice*’ (S15).

## Discussion

### Survey sample as a representation of larger population

The gender distribution among respondents (5% male, 93% female, 2% non-binary) roughly aligns with the Health and Care Professional Council’s (HCPC) Diversity Data Report (2021) which reported 8% men, 92% women, and 0% non-binary in the occupational therapy workforce and with the World Federation of Occupational Therapists’ Human resources project (2022), which reported 94% as the median number of occupational therapists that were female across 96 WFOT member countries. Over half (61%) of respondents identified as having a disability, health condition, specific learning disability, or as being neurodivergent, which was significantly higher than the 11% reported by the HCPC (2021). This difference is likely due to volunteer bias, as discussed later in the limitations section.

### Key themes

This research looked at how occupational therapy students view and experience ableism in their field. The results show a complicated situation. Students have different understandings of what ableism is, and they see it happening in both their classes and work placements. Encouragingly, a strong desire to challenge ableism and deepen their understanding of disability studies was evident.

#### Occupational Therapy Focus, Practice and Values

This study’s findings revealed several aspects of occupational therapy focus, practice, and values that warrant critical examination within the context of ableism. One of the core tenets of occupational therapy, an emphasis on independence (Kristensen, Præstegaard and Ytterberg, 2017), was overwhelmingly agreed upon by respondents. This emphasis on independence can inadvertently perpetuate ableist ideologies by prioritising normative standards of functioning (Hammell, 2021, 2022, 2023; Mahipaul, 2022; Yao et al., 2022). The profession’s focus on activities of daily living (ADL) also emerged prominently in the survey responses. While ADLs are undeniably crucial for many individuals, an overemphasis on these activities without sufficient consideration of individual needs and preferences may reinforce ableist assumptions about what constitutes meaningful occupation (Hammell, 2021, 2022, 2023; Mahipaul, 2022; Yao et al., 2022).

Moreover, the perception of occupational therapists as expert enablers and the tendency to prioritise the performance of occupations to a standardised norm were aspects of occupational therapy practice that resonated with a significant portion of respondents. While expertise is undoubtedly valuable, positioning occupational therapists as sole enablers risks disempowering clients and perpetuating paternalistic attitudes (Hammell, 2022). Similarly, the prioritisation of standardised norms may inadvertently marginalise individuals whose abilities and preferences fall outside these norms (Hammell, 2021, 2022, 2023; Mahipaul, 2022; Yao et al., 2022). In their open comments, respondents underscored the value of clients as experts in their own health and the necessity of their involvement in goal setting and treatment, emphasising these too are key factors in occupational therapy practice.

#### Understanding of Ableism and Perceptions of Ableism within Occupational Therapy

The survey results shed light on students’ understanding of ableism and its perceived manifestations within occupational therapy. A notable percentage recognised its presence within the profession. However, others disagreed with the assertion that occupational therapy is inherently ableist. This discrepancy can be attributed to several factors. Those who agreed often cited the profession’s emphasis on ‘normal’ function and standardised assessments, which can inadvertently exclude individuals who do not fit within societal norms. Conversely, those who disagreed often highlighted the profession’s focus on empowerment, potential for positive impact, and cited that it was dependent on both individual practitioners and different areas of practice. These findings suggest a complex interplay between the profession’s ideals and its potential for perpetuating ableist practices, highlighting the need for ongoing critical reflection and advocacy within occupational therapy.

A key finding was the variation in respondents’ definitions and interpretations of ableism, reflecting the multifaceted nature of the concept. While some emphasised ableism as a socially constructed notion of normalcy, others focused on discrimination or invalidation of disabled individuals. This diversity of perspectives underscores the importance of engaging in ongoing dialogue and critical reflection to deepen understanding and address ableism effectively.

While the study provided valuable insights into the relationship between awareness and education on perceptions of ableism in occupational therapy, it is important to acknowledge the limitations of its size and complexity. The findings suggested that increasing awareness and understanding of ableism can prompt critical reflection and reshape perspectives within the profession, but further research is warranted to confirm and generalise these results. A larger-scale study with a more diverse sample could provide a more comprehensive understanding of this issue.

#### Are things beginning to change?

This study’s findings highlight both challenges and opportunities for addressing ableism within occupational therapy. While a proportion of students reported encountering or experiencing ableism, there were also indications of a desire to challenge and dismantle ableist attitudes and practices. However, the perceived level of institutional support for challenging ableism varied widely among respondents, with concerns raised about potential repercussions for speaking out.

There are indications within this study’s results that change may already be beginning. Respondents’ demographic data showed a much higher percentage of disabled, neurodivergent, or those with long-term health conditions compared to figures in the latest HCPC (2021) workforce report. There are several potential explanations for this. It could be due to volunteer bias, please see limitations section, or it could be that respondents within this anonymous survey were more comfortable disclosing this information than professionals responding to a workforce survey. However, it could also indicate that the profession is beginning to attract a more diverse cohort of students, potentially more representative of the people the profession serves, aligning with more general healthcare commitments to promote representation within the workforce (Wilbur et al., 2020).

Another indication from this study that the profession is in a potential period of change is that more students reported the use of the medical model within practice than within education. This could indicate that although the medical model is still widely seen in practice, it is losing popularity within educational spheres. Therefore, as new occupational therapists enter the workforce, they may bring with them new ways of considering disability.

A founding member of ABLEOT UK (2021), an occupational therapy network/advocacy group, recently called on the Royal College of Occupational Therapists (RCOT) to review their accreditation process to ‘*ensure newly qualified OTs . . . do not enter the workplace with ableist, racist, homophobic and transphobic views*’ (Hicks, 2022). Results from this study indicate that students are aware of the internal biases they may hold around ableism and overwhelmingly would welcome additional support to engage with disability studies. However, Grenier (2020, p. 640) warns that by focusing on recognising biases the burden is placed ‘*on individuals to ‘fix the problem’, rather than on institutions . . . to radically dismantle and rebuild the ‘biased’ frameworks and models . . .*’

Despite these potential positive shifts, efforts to combat ableism within occupational therapy must be multifaceted and sustained. This includes integrating disability studies more comprehensively into occupational therapy education, fostering environments where students feel empowered to challenge ableism, and promoting reflexivity among practitioners to recognise and address unconscious biases.

### Limitations

#### Volunteer bias

This study relied on volunteers, which can influence the results (Sedgwick, 2013). People with a strong interest in ableism or disability studies may have been more likely to participate, potentially overrepresenting certain perspectives.

#### Social desirability bias

Participants might have felt pressure to give answers they thought were considered favourable, rather than their true experiences or opinions (Brenner and DeLamater, 2016). While anonymity may have helped reduce potential bias, it is worth considering when interpreting the reported behaviours and attitudes.

##### Researcher bias

Researchers’ own beliefs can unconsciously influence how they design a study and interpret the results. To address this, the research team continuously reflected on their biases throughout the study (Johnson et al., 2020). Additionally, the authors’ diverse backgrounds in occupational therapy experience, disability, health conditions, and neurodivergence helped minimise individual biases and strengthen the study’s overall validity.

##### Survey design limitations

As a survey-based study, this research was limited by its reliance on self-reported data and the inability to delve deeply into participants’ individual experiences. Additionally, the survey format may have constrained respondents’ ability to express complex or nuanced perspectives.

A statement regarding ableism within the curriculum was removed due to unclear wording and varying interpretations by participants. This highlights the importance of clear and concise survey design. Additionally, comments made by respondents regarding occupational therapists as ‘expert enablers’ highlighted inconsistencies in their understanding of the term enabling. While this question was included in the results, it is important to note that the term ‘enabling’ was interpreted in various ways by different participants. For example, some respondents viewed enabling as empowering individuals with disabilities, while others saw it as a form of paternalism. These differing perspectives underscore the need for further exploration and clarification of the concept of ableism within the field of occupational therapy. Future research could delve into the factors that influence these varying interpretations, such as age, gender, cultural background, or educational experiences.

## Conclusion

This study explored occupational therapy students’ perspectives on ableism. The findings reveal a complex landscape. While some students hold a clear understanding of ableism and readily identify its presence in occupational therapy, others express differing interpretations or even disagreement.

The emphasis on independence and standardised norms in occupational therapy practice emerged as potential areas for critical examination. These goals can inadvertently reinforce ableist assumptions about what constitutes a meaningful life and marginalise individuals whose abilities or preferences differ from the norm.

There is, however, hope for change. The student body appears to be diversifying, potentially leading to a workforce that better reflects the population it serves. Furthermore, a strong desire to challenge ableism and deepen understanding of disability studies was evident among the students.

Moving forward, occupational therapy education should integrate disability studies more comprehensively. Additionally, fostering environments where students feel empowered to challenge ableism and promoting reflexivity among practitioners are crucial steps. By acknowledging and addressing ableist biases, the occupational therapy profession can ensure its practices are truly inclusive and empower individuals to participate meaningfully in all aspects of life.

**Key findings**

- Thirty-four percent of student respondents perceived occupational therapy as ableist.
- Sixty-three percent of student respondents reported witnessing and/or experiencing ableism during their practice placements.
- Ninety-four percent of student respondents agreed they would like more support to engage in disability studies.

**What the study as added**

This study adds to the evidence-base regarding occupational therapy students’ perceptions of ableism within occupational therapy education and practice and informs future efforts to create a more inclusive profession.
